# Case Report: Compound heterozygous KCTD7 variants in two siblings presenting with myoclonic epilepsy and ataxia

**DOI:** 10.3389/fnins.2025.1670008

**Published:** 2025-11-12

**Authors:** Jingjing Song, Wenlin Wu, Yang Tian, Luoxiao Qin, Shitao Wei, Bin Yu, Hao Su, Liping Huang, Wenhui Liu, Xiaoli Huang

**Affiliations:** 1 Department of Neurology, Liuzhou Hospital, Guangzhou Women and Children’s Medical Center, Liuzhou, China; 2 Department of Neurology, Guangzhou Women and Children’s Medical Center, Guangzhou Medical University, Guangzhou, China

**Keywords:** KCTD7 gene, myoclonic epilepsy, ataxia, whole-exome sequencing, children

## Abstract

**Objective:**

Biallelic variants in *KCTD7* have been associated with progressive myoclonic epilepsy (PME), a rare autosomal recessive disorder characterized by early-onset epilepsy, cognitive decline, myoclonus, and ataxia.

**Methods:**

Whole-exome sequencing was first performed in the elder sister to identify candidate variants, followed by in silico pathogenicity prediction. Sanger sequencing was then used to validate the variants in both parents and the younger brother.

**Results:**

We report two siblings with progressive myoclonic epilepsy (PME) carrying compound heterozygous *KCTD7* variants: c.334C > T (p.Arg112Cys), a paternally inherited variant previously reported in homozygous form and currently classified as likely pathogenic, and c.640C > T (p.Arg214Trp), a novel maternally inherited variant currently classified as of uncertain significance. Both patients presented between 2 and 3 years of age with gait instability, myoclonic seizures, and developmental regression. EEG revealed background slowing, multifocal spike–slow wave discharges, and electrical status epilepticus during sleep. Brain MRI findings were initially unremarkable despite progressive neurological deterioration. Whole-exome sequencing and Sanger validation confirmed the variants and their segregation. In silico tools predicted both variants to be deleterious, and structural modeling using PyMOL and I-Mutant 3.0 demonstrated that both variants likely disrupt local residue interactions and reduce protein stability. Both patients received antiepileptic therapy and immunomodulatory treatment, including intravenous methylprednisolone and immunoglobulin. The proband achieved seizure control and improved gait following immunotherapy, though cognitive deficits persisted. The younger sibling exhibited a more severe disease course, with progressive cognitive decline, speech and visual impairment, and loss of independent ambulation, despite partial seizure control. These findings expand the genetic and phenotypic spectrum of KCTD7-related PME and suggest that immunotherapy may confer partial clinical benefit in selected cases.

**Conclusion:**

This case expands the variant spectrum of *KCTD7*-related disorders and emphasizes the utility of comprehensive genetic testing in early-onset neurodegenerative epileptic syndromes. Functional studies are needed to clarify the clinical significance of the novel *KCTD7* variant.

## 1 Introduction

*KCTD7* encodes a highly conserved BTB/POZ-domain-containing protein predominantly expressed in the human brain, including cortical neurons, hippocampal pyramidal and granular layers, cerebellar Purkinje cells, and corticospinal tracts (Blumkin et al., 2012). Although *KCTD7* does not form potassium channels directly, it may regulate neuronal excitability via interactions with voltage-gated potassium channel subunits and cullin-RING E3 ligase complexes (Blumkin et al., 2012). Loss-of-function variants in *KCTD7* disrupt neuronal membrane polarization and autophagy-related pathways, contributing to progressive neurodegeneration and epileptogenesis (Blumkin et al., 2012).

Biallelic *KCTD7* variants are a known cause of autosomal recessive progressive myoclonic epilepsy type 3 (PME3), characterized by early-onset drug-resistant epilepsy, myoclonic and generalized seizures, progressive cognitive decline, and ataxia (Gil-Nagel et al., 2019). First reported in a consanguineous family in 2007, *KCTD7*-related PME remains rare, with limited case numbers and variant spectrum (Blumkin et al., 2012).

Here we report two siblings from a non-consanguineous family carrying compound heterozygous *KCTD7* variants, including a previously unreported missense variant. Both presented with early-onset myoclonic epilepsy, ataxia, cognitive regression, and electroclinical features consistent with PME. This case expands the variant spectrum of *KCTD7* and emphasizes the importance of molecular diagnostics in children with developmental regression and refractory seizures.

### 1.1 Case description

#### 1.1.1 Patient 1 (Proband)

The proband, a girl born at term following an uncomplicated vaginal delivery, was the first child of non-consanguineous healthy Han Chinese parents. Her birth history was unremarkable, and early developmental milestones were achieved appropriately. There was no known family history of epilepsy or other neurological disorders. At age 3 years 8 months, she developed gait instability with dysarthria. One month later, she developed seizures, including generalized tonic–clonic, myoclonic, and focal seizures with impaired awareness (chewing automatisms). Initial evaluations and management were performed at local hospitals. Levetiracetam (titrated to 30 mg/kg/day) was initiated with partial improvement. After 5 months, gait instability worsened, with nocturnal seizures characterized by staring, clenched fists, upper limb tremor, and lower limb tonic posturing lasting ~1 min. Levetiracetam was increased (44 mg/kg/day), and low-dose levodopa (3 mg/kg/day) was empirically added, resulting in transient motor improvement. Three months later, she experienced additional prolonged seizures and progressive gait deterioration. At age 6 years 5 months, she was referred to our institution for further assessment due to persistent gait instability and recurrent seizures. Neurological examination at admission revealed dysarthria, normal muscle strength and tone, unstable standing and walking. Cerebellar signs were present, including positive finger-to-nose, heel-to-shin, and rapid alternating movement tests.

**Figure 1:**
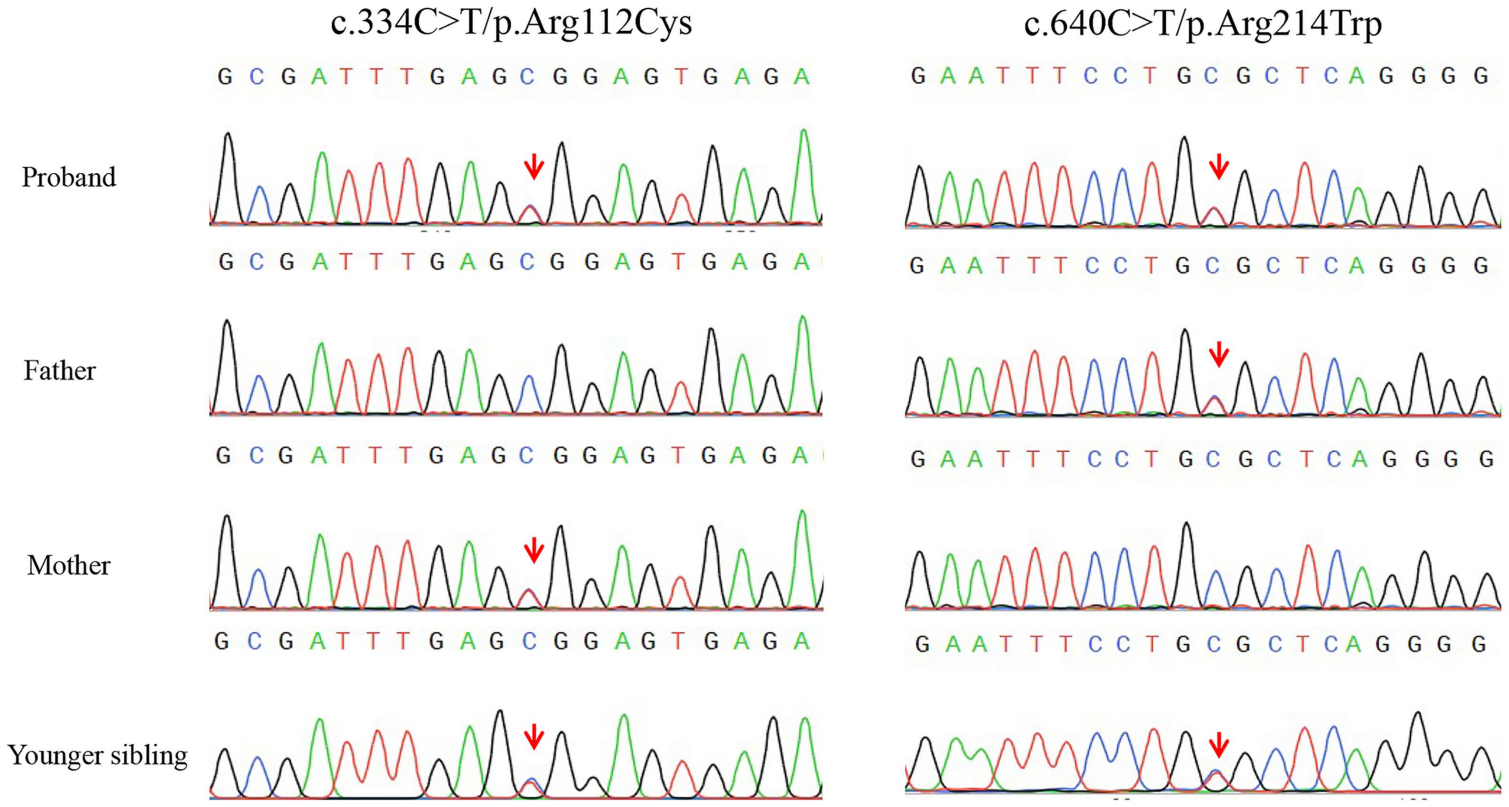
Sanger sequencing confirmation of compound heterozygous KCTD7 variants in the proband and her family members. Two heterozygous variants were identified: c.334C > T (p.Arg112Cys) in exon 3 (left) and c.640C > T (p.Arg214Trp) in exon 4 (right). In the proband, both variants are present in the heterozygous state, indicated by overlapping peaks at the respective sites (red arrows). The father carries the heterozygous c.334C > T variant, while the mother carries the heterozygous c.640C > T variant. The younger sibling also carries both heterozygous variants, consistent with the same compound heterozygous genotype as the proband.

**Figure 2:**
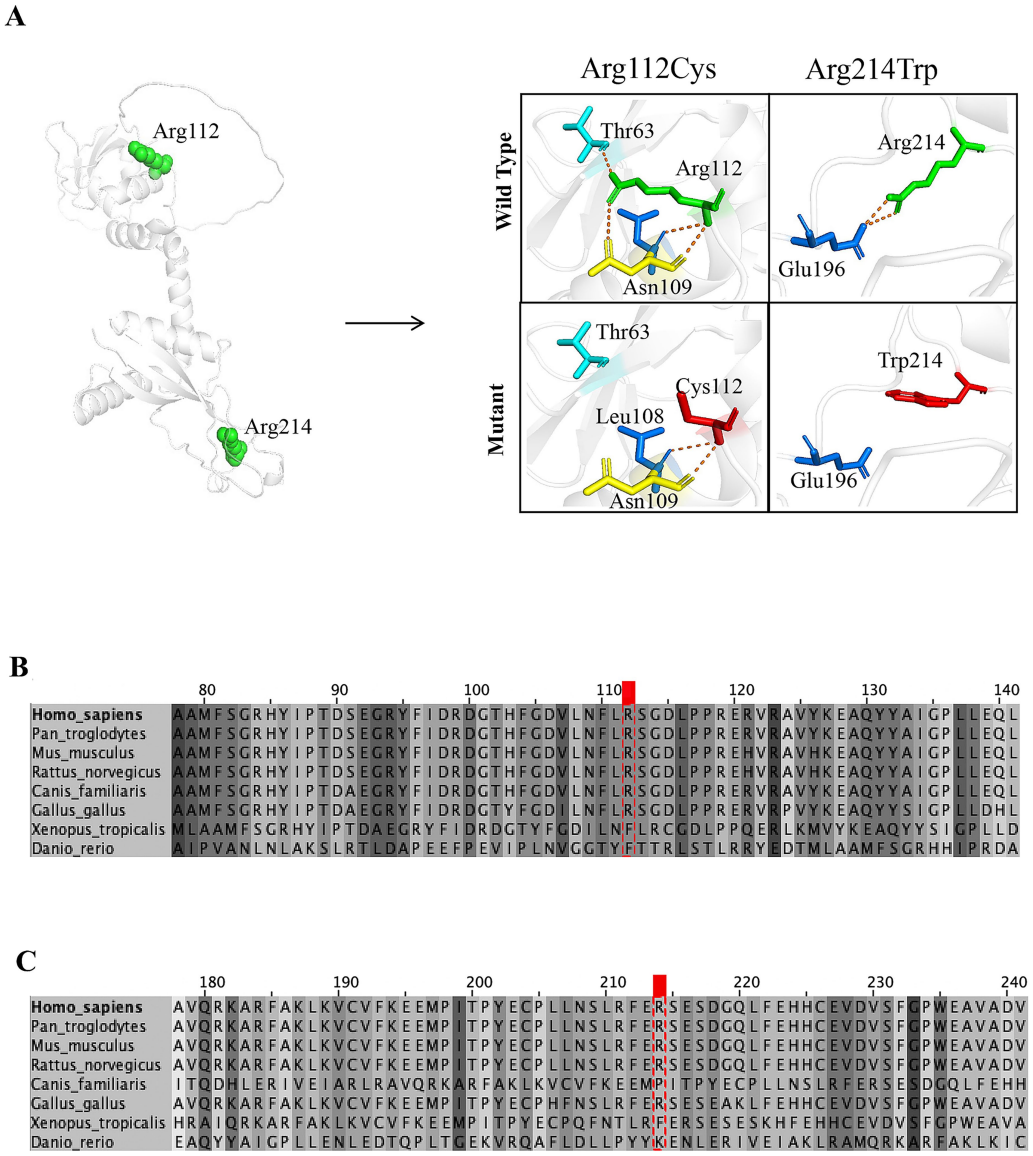
Structural modeling and evolutionary conservation of KCTD7 missense variants. **(A)** Structural modeling of the KCTD7 variants p.Arg112Cys and p.Arg214Trp. The left panel shows the spatial positions of Arg112 and Arg214 in the 3D structure of KCTD7. The right panels depict local interactions of the wild-type residues (top) and the mutant residues (bottom). In the wild-type structure, Arg112 (green) forms hydrogen bonds with Thr63 and Asn109; substitution with Cys (red) disrupts these interactions and introduces a new contact with Leu108. Similarly, Arg214 (green) interacts with Glu196, which is lost upon substitution with Trp (red), potentially destabilizing the local structure. Hydrogen bonds are shown as dashed orange lines. Structural visualization was performed using PyMOL 2.0, and mutation-induced stability changes were predicted with I-Mutant 3.0. **(B,C)** Multiple sequence alignment of the protein regions containing the variants. Alignments were performed using Clustal Omega in Jalview (version 2.11.4.1). Species and reference sequence accession numbers are: *Homo sapiens* (AAH42482), *Pan troglodytes* (XP_016813036), *Mus musculus* (NP_766097.1), *Rattus norvegicus* (NP_001121666), *Canis familiaris* (XP_038523730.1), *Gallus gallus* (NP_001034358), *Xenopus tropicalis* (XP_004911762), *Danio rerio* (NP_001038798.2). The red box marks the variant site in the human sequence; shading intensity indicates residue conservation across species. **(B)** Variant p.Arg112Cys. **(C)** Variant p.Arg214Trp.

**Figure 3:**
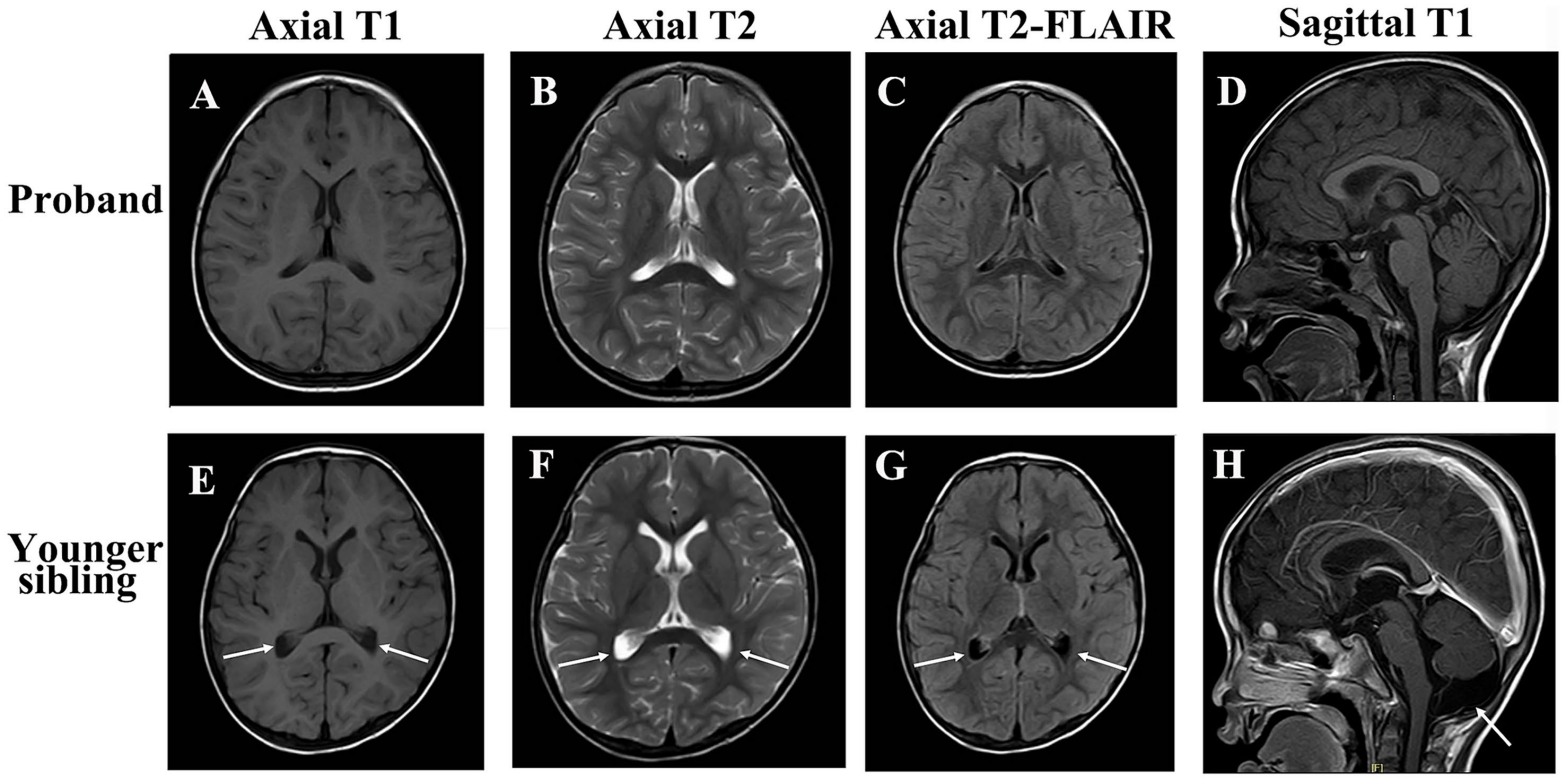
Brain MRI of the patients. Brain MRI of the proband: **(A)** Axial T1-weighted image; **(B)** Axial T2-weighted image; **(C)** Axial T2-weighted FLAIR image. **(D)** Sagittal T1-weighted No structural abnormalities were observed. Brain MRI of the proband’s younger brother: **(E)** Axial T1-weighted image; **(F)** Axial T2-weighted image; **(G)** Axial T2-weighted FLAIR image. **(H)** H Sagittal T1 post-contrast; Mild lateral ventricular enlargement and a prominent cisterna magna were noted. Arrowheads in **(E–G)** indicate mild lateral ventricular enlargement; the arrow in (H) highlights a prominent cisterna magna.

Diagnostic investigations were obtained at different time points during the disease course. Whole-exome sequencing identified compound heterozygous variants in *KCTD7*: c.334C > T (p.Arg112Cys) in exon 3, inherited from her father, and c.640C > T (p.Arg214Trp) in exon 4, inherited from her mother. The variants were subsequently confirmed by Sanger sequencing in both parents. The c.334C > T (p.Arg112Cys) variant had previously been reported as a homozygous variant in a patient with *KCTD7*-related progressive myoclonic epilepsy (Yoganathan et al., 2024). According to ClinVar, this variant was classified as uncertain significance (VUS) under record RCV001297140.8 (last evaluated June 22, 2022), but was subsequently reclassified as likely pathogenic (LP) under record RCV001562390.4 (last evaluated March 5, 2024). The c.640C > T (p.Arg214Trp) variant, inherited from the mother, has not been reported in the literature. However, an entry for this variant was submitted to ClinVar under record RCV001065036.8, where it is currently classified as a variant of uncertain significance (VUS) (last evaluated February 8, 2022). This variant affects a highly conserved residue and is predicted to be deleterious by multiple in silico algorithms. Both variants are rare (minor allele frequency <0.0005 in gnomAD). Sanger sequencing confirmed segregation of the variants within the family (Figure 1). Protein structural modeling using PyMOL and I-Mutant 3.0 demonstrated that both missense variants are likely to disrupt local residue interactions and reduce protein stability, supporting their pathogenic potential (Figure 2A). Multiple sequence alignment showed that the affected residues are highly conserved across vertebrate species, including *Homo sapiens*, *Pan troglodytes*, *Mus musculus*, *Rattus norvegicus*, *Canis familiaris*, *Gallus gallus*, *Xenopus tropicalis*, and *Danio rerio*. The variant sites are highlighted in red, and shading intensity reflects the degree of residue conservation (Figures 2B,C). According to American College of Medical Genetics and Genomics (ACMG) criteria, the c.334C > T variant can be classified as likely pathogenic, supported by rarity in population databases (PM2: absent or extremely rare in population databases), deleterious computational predictions (PP3: multiple in silico algorithms support a damaging effect), and segregation evidence in the family (PP1: cosegregation with disease in affected family members), while the c.640C > T variant remains a variant of VUS. Laboratory investigations were unremarkable, including blood lead level, cerebrospinal fluid (CSF) routine and biochemistry, autoimmune encephalitis antibody panel (NMDAR, AMPAR1/2, LGI1, CASPR2, GABABR, IgLON5, DPPX, GAD65, GlyRα1, mGluR5, and D2R in both serum and CSF), cerebellar ataxia–related antibodies (GAD65, Homer3, ARHGAP26, ATP1A3, CARP VIII, NCDN, GluRδ2, CASPR2, PCA2, Yo, mGluR1/2/8, and KLHL11 in serum and CSF), and paraneoplastic neuronal antibody panel (Hu, Ri, CV2, Ma1/2, amphiphysin, GAD65, Tr, Zic4, PKCγ, and SOX1 in CSF). Brain and spinal magnetic resonance imaging (MRI) were normal (Figures 3A–D). Video EEG demonstrated generalized background slowing with frequent frontally predominant spikes, slow waves, and spike-slow wave discharges during both wakefulness and sleep. Cognitive evaluation indicated intellectual disability (full-scale IQ 61; verbal IQ 58; performance IQ 69) and moderate social adaptation impairment.

A diagnosis of KCTD7-related epilepsy, presenting with generalized and focal seizures as well as ataxia, was considered. Levodopa was discontinued, and combination therapy with clonazepam (1 mg nightly), valproate (15 mg/kg/day), and levetiracetam (35 mg/kg/day) achieved partial seizure control while gait instability persisted. Subsequent video electroencephalography (EEG) revealed electrical status epilepticus in sleep (ESES) with a spike–wave index of 85–90%. Immunotherapy was initiated, including intravenous methylprednisolone (20 mg/kg/day, followed by oral taper) and intravenous immunoglobulin (initial 2 g/kg over 4 days, followed by monthly 400 mg/kg infusions for 4 months). Seizures resolved, and gait instability improved following treatment. At the last follow-up (age 8 years 9 months), she was able to walk independently without seizures; however, cognitive impairment remained. EEG continued to show frequent right frontal-central-parietal spike discharges during sleep.

#### 1.1.2 Patient 2 (Younger brother)

The proband’s younger brother was the second child of the same non-consanguineous healthy Han Chinese parents. His birth history was unremarkable, with normal early developmental milestones. There was no known family history of epilepsy or neurological disorders.

At age 1 year 5 months, he developed a febrile episode (exact temperature unknown) accompanied by a single seizure characterized by loss of consciousness and generalized tonic stiffening lasting approximately 2 s, without cyanosis, foaming, or incontinence. Following recovery, he appeared mentally alert but developed bilateral hand tremors lasting several seconds, occurring several to dozens of times daily.

At age 2 years 1 month, he experienced another febrile seizure with similar semiology. Approximately one week after defervescence, gait instability emerged, characterized by frequent falls, intermittent head shaking, and worsening hand tremors (approximately 20 episodes per day, each lasting about 10 s). He was admitted to a local hospital for evaluation. Brain MRI was unremarkable. Video EEG demonstrated background slowing with medium- to high-amplitude 2–2.5 Hz spike-slow wave complexes predominantly in the bilateral occipital and posterior temporal regions during sleep. For Patient 2, genetic testing was performed by targeted Sanger sequencing only, due to family preference and financial constraints. This analysis confirmed the same compound heterozygous *KCTD7* variants as identified in his sister. A presumptive diagnosis of *KCTD7*-related myoclonic epilepsy with ataxia was made. Valproic acid was initiated at 20 mg/kg/day, resulting in seizure control. At age 2 years 8 months, he developed dysarthria, drooling, and instability in standing, walking, and sitting. At 3 years 11 months, new seizure episodes appeared, characterized by head drops lasting several seconds and resolving spontaneously. Follow-up video EEG revealed background slowing, bilateral posterior spike-slow wave discharges, and absent sleep spindles, with predominance in the occipital region. Clonazepam was added at 0.08 mg/kg/day for seizure control.

At 4 years 8 months, he presented with visual impairment, manifested by searching for objects by tactile exploration and only recognizing large, brightly colored objects within 50 cm. His speech became increasingly unclear, reactions slowed, and gait instability worsened, though no seizures were reported during this period.

Upon admission to our institution, ophthalmologic evaluation showed right eye visual acuity of 0.6 and left eye refraction of −1.25/−0.25 × 1.18. A clinical phenotype consistent with PME was observed, characterized by myoclonic seizures, ataxia, and neurodevelopmental regression, although generalized tonic–clonic seizures were not clearly documented. Brain MRI demonstrated mild lateral ventricular enlargement and a prominent cisterna magna (Figure 3E–H). Treatment included intravenous methylprednisolone (20 mg/kg/day, followed by gradual oral taper), intravenous immunoglobulin (initial dose 2 g/kg over 4 days), and continued antiepileptic therapy with valproic acid and clonazepam. Levetiracetam was subsequently added and titrated to 40 mg/kg/day. Repeat video EEG demonstrated background slowing with continuous right hemispheric spike-slow wave and polyspike-slow wave discharges, with a spike–wave index of 75–85%.

At the last follow-up (age 5 years and 2 months), he was unable to stand or walk independently. Drooling persisted, though no seizures were observed. Physical examination showed alertness with cognitive decline, inability to follow simple commands, unstable sitting and head control, lower limb strength grade 4, upper limb strength grade 3+, and generalized hypotonia. Repeat video EEG demonstrated abnormal school-age EEG with poorly distinguishable sleep–wake stages, showing continuous widespread low- to medium-amplitude spikes, spike-slow waves, spikes, and polyspike-slow wave discharges, predominantly over the right hemisphere, with a spike–wave index of approximately 85–95%.

### 1.2 Discussion

Previous studies have shown that KCTD7 encodes a BTB/POZ-domain protein that interacts with potassium channel subunits and cullin-RING E3 ligase complexes, thereby regulating neuronal excitability and autophagy pathways (Mastrangelo et al., 2019; Moen et al., 2016; Richards et al., 2015). Loss-of-function variants result in neuronal hyperexcitability and impaired autophagy, providing a mechanistic basis for progressive neurodegeneration and seizures observed in affected patients. Clinically, KCTD7-related PME has been reported in approximately 40 patients worldwide, typically with onset in early childhood, presenting with myoclonic or generalized seizures, ataxia, and progressive cognitive decline (Azizieh et al., 2011).

Building on this background, we describe two siblings from a non-consanguineous family carrying compound heterozygous variants in KCTD7: c.334C > T (p.Arg112Cys) and c.640C > T (p.Arg214Trp). The c.334C > T variant, inherited from the father, has been previously reported in homozygous form in patients with KCTD7-related PME and is currently classified as likely pathogenic (ClinVar records RCV001297140.8 and RCV001562390.4). Functional studies have suggested that this variant impairs binding to cullin-3 E3 ligase complexes, leading to lysosomal and autophagic dysfunction, consistent with progressive neurodegeneration (Alsini et al., 2023). The c.640C > T (p.Arg214Trp) variant, inherited from the mother, has not been reported in the literature but was submitted to ClinVar (RCV001065036.8), where it remains classified as a variant of uncertain significance (VUS). This variant affects a highly conserved residue and is predicted to be deleterious by multiple in silico tools. In line with ACMG criteria, the c.334C > T variant can be considered likely pathogenic (supported by PM2, PP3, and PP1), whereas the c.640C > T variant remains a VUS (Burke et al., 2021). Of note, p.Arg112Cys lies within the BTB/POZ domain (amino acids ~51–149), a region critical for CUL3 binding and protein–protein interactions. Variants within this domain, such as p.Arg94Trp, have previously been shown to reduce membrane expression, and other nearby variants impair CUL3 binding, supporting the functional relevance of this region (Farhan et al., 2014). By contrast, p.Arg214Trp is located outside known functional domains. Although no specific function has been assigned to this region, the residue is highly conserved, and structural modeling predicts loss of stabilizing interactions, suggesting potential pathogenicity despite the absence of domain-level annotation. We acknowledge that we could not empirically validate these effects in this study and have listed this as a limitation.

Both siblings presented with ataxia, cognitive decline, and myoclonic seizures. EEG showed multifocal epileptiform discharges, background slowing, and sleep-activated discharges including ESES with spike–wave indices of 85–95%. MRI findings were unremarkable, which aligns with previous literature indicating nonspecific or absent MRI abnormalities in KCTD7-related PME. These clinical and electroencephalographic findings support the role of KCTD7 dysfunction in cortical hyperexcitability, likely mediated through potassium channel dysregulation and impaired autophagy support the role of KCTD7 dysfunction in cortical hyperexcitability, likely through potassium channel dysregulation and impaired autophagy (Yoganathan et al., 2024).

Treatment response in KCTD7-related PME is often poor (Alsini et al., 2023). In our patients, levetiracetam, valproic acid, and clonazepam provided partial seizure control, with GABAergic agents appearing more effective than sodium channel blockers. The transient motor improvement observed after immunomodulatory treatment (IVIG and steroids) raises the possibility of non-specific anti-inflammatory or neuroprotective effects, although autoantibody testing was negative. Notably, seizures often become refractory, with most patients requiring multiple ASMs. Despite seizure reduction, disease progression remains largely unaltered, highlighting the urgent need for targeted therapeutic strategies. Proposed future directions include functional validation of novel variants, modeling with patient-derived iPSC neurons, and exploration of downstream pathways such as mTOR or autophagy activation for therapeutic targeting (Yoganathan et al., 2024).

Evidence for immunotherapy in KCTD7-related disease is limited and case-based. In the original consanguineous family (three affected children), short courses of hydrocortisone (5 mg/kg/day) in two patients produced marked but transient benefit after failure of antiseizure medications (Blumkin et al., 2012). An additional opsoclonus–myoclonus–ataxia–like case showed sleep “continuous spike–wave” without SWI reporting and responded to steroids (Metz et al., 2018). In a multicenter cohort of developmental and/or epileptic encephalopathy with spike–wave activation in sleep (*n* = 91), one child carried KCTD7, supporting a link to sleep-activated spike–wave activation, although individual treatment details were not provided (Van Bogaert et al., 2007). In the largest KCTD7 series to date (42 cases), sleep-activated generalized and multifocal epileptiform discharges were common and sometimes resembled spike–wave activation in sleep, but ESES was not explicitly diagnosed and SWI values were not reported (Alsini et al., 2023). In our two siblings with quantified ESES (SWI 85–95%), combined intravenous methylprednisolone and intravenous immunoglobulin were administered, and seizures were suppressed with improved gait in one patient, whereas the other showed no clear benefit. Collectively, steroids/IVIG may dampen sleep-potentiated epileptiform activity in a subset of KCTD7 patients, but effects appear partial and frequently temporary. Given that KCTD7 loss mainly perturbs neuronal excitability and autophagy–lysosome pathways rather than primary immunity, any immunotherapy benefit is likely symptomatic/network-level (anti-inflammatory) rather than disease-modifying, underscoring the need for prospective, genotype-annotated studies (Yoganathan et al., 2024).

The novel c.640C > T (p.Arg214Trp) mutation expands the known variant spectrum of KCTD7. This case underscores the importance of comprehensive genetic testing in early-onset neurodegenerative epilepsy, particularly when imaging is non-diagnostic. Early molecular diagnosis facilitates family counseling, rapid diagnosis in siblings, and offers options for prenatal or preimplantation genetic diagnosis.

The findings in our family demonstrate the typical course of *KCTD7-*related PME, with early-onset ataxia, refractory epilepsy, cognitive regression, ESES on EEG, and minimal MRI findings. Our report contributes novel genotype–phenotype data and emphasizes the clinical significance of *KCTD7* mutations in PME.

This study has certain limitations. Whole-exome sequencing successfully identified compound heterozygous variants in *KCTD7*, but it does not detect all possible genetic causes of PME. Pathogenic variants in the mitochondrial genome (e.g., *MT-TK* in MERRF) or repeat expansions (e.g., *FXN* in Friedreich ataxia) would not be captured by this approach. In our patients, the clinical phenotype was highly consistent with *KCTD7*-related PME, and no other candidate variants were identified on WES, making alternative diagnoses unlikely. Nevertheless, the absence of additional testing such as mitochondrial sequencing or repeat expansion analysis remains a limitation of the present report. Additionally, despite the availability of patient specimens, we could not perform complementary molecular assays due to current laboratory resource limitations. These important validations are therefore deferred to future studies.

## 2 Conclusion

This case expands the variant spectrum of *KCTD7*-related disorders and emphasizes the utility of comprehensive genetic testing in early-onset neurodegenerative epileptic syndromes. Functional studies are needed to clarify the clinical significance of the novel *KCTD7* variant.

## Data Availability

The datasets presented in this article are not readily available because of ethical and privacy restrictions. Requests to access the datasets should be directed to the corresponding author/s.
